# Different Occlusal Schemes in a Persistent Protruding Complete Denture Wearer

**DOI:** 10.1155/2016/7418686

**Published:** 2016-03-16

**Authors:** Carolina Mayumi Iegami, Danilo de Melo Lopes, Atlas Edson Moleros Nakamae, Priscila Nakasone Uehara, Regina Tamaki

**Affiliations:** Department of Prosthodontics, School of Dentistry, University of Sao Paulo, Avenida Professor Lineu Prestes, 2227 Cidade Universitaria, 05508-000 Sao Paulo, SP, Brazil

## Abstract

Different types of artificial teeth and occlusal designs can be used in complete dentures. Bilateral balanced occlusion, lingualized occlusion, canine guidance, and monoplane are the main occlusal designs; however there is no agreement on which tooth arrangement is ideal for achieving success in complete dentures. This report presents an alternative for persistent involuntary protruding complete denture wearers through the use of artificial teeth with higher cusps. Due to an old and worn pair of complete dentures, the patient had the habit of protruding. New dentures were made with Biotone artificial teeth and in the trial session, the patient would still protrude. A new set was made with Premium artificial teeth, which present higher cusps. With these dentures, the involuntary protrusion did not occur. From the delivery to the follow-up sessions, the patient stopped protruding.

## 1. Introduction

Despite the efforts to reduce tooth loss in the world, edentulism prevails in most countries, presenting higher rates, especially in countries where poverty exists [1]. In these countries, complete dentures are the primary choice of the edentulous patients for cost reasons and most patients, while satisfied, do not replace their dentures in the recommended period.

Long-time use of the same pair of complete dentures might lead to aesthetics impairment, loss of vertical dimension, and reduction of masticatory efficiency [2, 3]. Since complete dentures are mucosa-supported, they require stability on the support area to function properly. Therefore, occlusion is a key component for denture stability [4]. Although complete dentures have been used in prosthodontics for over a hundred years, there is no consensus on which tooth arrangement is ideal for achieving success in complete dentures [5, 6]. Among the various commercial brands available, posterior teeth can present different morphology, being anatomical or monoplane. Anatomical posterior teeth are similar to the natural posterior teeth and can present different cusp angles and cusp heights, improving masticatory efficiency [5]. Bilateral balanced occlusion provides comfort for the patient, protects the tissues, and improves retention [7]. Canine-guided occlusion presents good retention and masticatory performance levels [5]. Lingualized balanced occlusion also provides comfort, retention and is the occlusion choice for patients with residual ridge resorption [8, 9]. Monoplane posterior teeth are flat and were developed to minimize horizontal forces and improve stability [10].

According to Hanau, artificial dental cusps height may vary according to the inclination of the condylar guidance. The higher the inclination of the condylar guidance is, the higher the artificial teeth cusps can be [11]. Height of cusps also improves esthetics and may please the patient, since they are similar to natural teeth [12, 13]. Therefore, despite the little options available in the market, some patients need teeth with different cusps heights, according to their condylar guidance inclination.

In theory, teeth with higher cusps should allow better comminution of food, since their smaller area of contact with the food promotes greater penetrating power into the food bolus; however, it has not yet been proven [4].

This clinical report displays how the application of different posterior teeth cusps heights can influence the excursive movements of the patient.

## 2. Case Presentation

This study was submitted to the University of Sao Paulo, School of Dentistry Ethics Committee under the protocol 90/2010 and approved FR330287.

**Figure 1:**
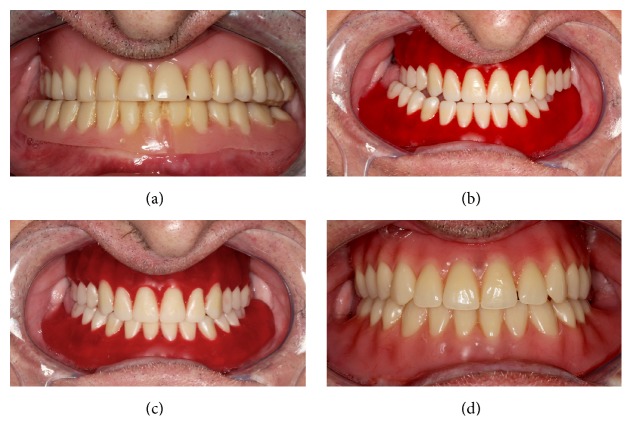
(a) Old complete dentures. (b) Repetitive movement with Biotone artificial teeth. (c) Trial complete dentures with Biotone artificial teeth. (d) Complete dentures with Premium artificial teeth.

A 59-year-old man, complete denture wearer for over 10 years, presented severe occlusal wear in the premolar and molar areas, vertical dimension was reduced, and the denture bases were ill adapted. Due to the posterior teeth wear, the patient was able to generate excursive movements freely (Figure 1(a)).

Given that the patient's complaint was that the dentures were old and teeth were abraded, new complete dentures were suggested as treatment.

Preliminary maxillary and mandibular impressions were made with modeling plastic impression compound (Godibar Placas; Lysanda Produtos Odontologicos Ltda, Brazil). Custom trays (JET; Artigos Odontologicos Classico Ltda, Brazil) were fabricated by taking reference of anatomical landmarks present in the diagnostic casts. Then, final casts were made with an impression with custom trays and a zinc-enolic paste (Pasta Lysanda; Lysanda Produtos Odontologicos Ltda, Brazil). Maxillary and mandibular wax rims were made with trial denture bases according to the esthetics requirements and vertical dimension was reestablished. Horizontal maxillomandibular record presented to be difficult as a result of the previous reduced vertical dimension and the posterior teeth wear.

Among the available techniques for the horizontal maxillomandibular record, the Paterson technique was chosen. In this technique, a mixture of carborundum and plaster is used to individualize the plane of occlusion and to promote neuromuscular disarrangement, allowing the horizontal maxillomandibular record.

Occlusal rims were mounted in an articulator (Articulador A7 Plus; Bio-Art Equipamentos Odontológicos Ltda, Brazil) with a facebow transfer and occlusal rims were fixed with metal staples.

Teeth chosen for the complete dentures were the Biotone (BT) (Dentsply International, Pennsylvania, USA) and the chosen occlusal scheme was the bilateral balanced occlusion. In the trial session, an unstable mandibular position was observed. The patient protruded repetitively, promoting the movement of the artificial teeth (Figures 1(b) and 1(c)).

**Figure 2:**
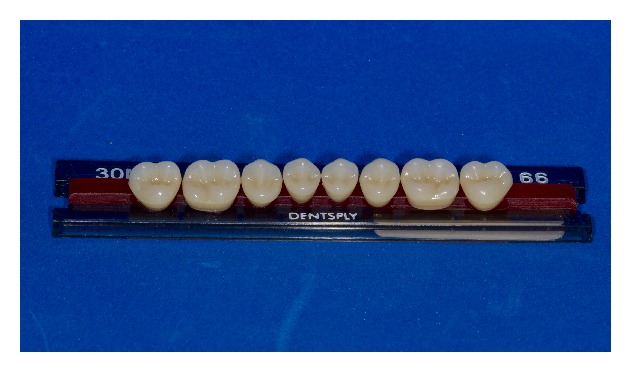
Biotone artificial teeth.

**Figure 3:**
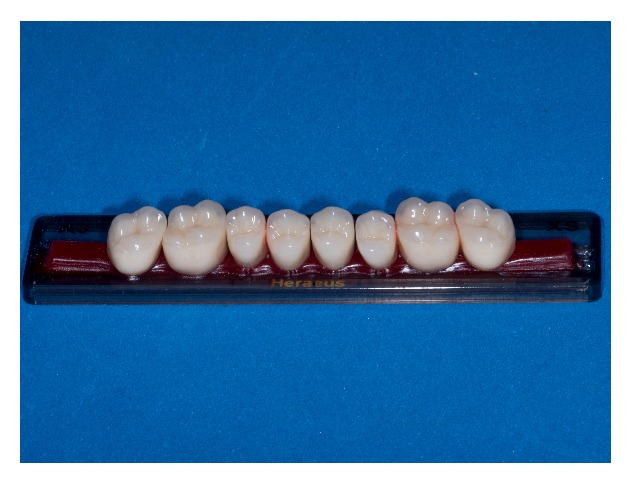
Premium artificial teeth.

A second pair of complete dentures was made with Premium (PT) (Heraeus Kulzer GmbH/Hanau, Germany) artificial teeth, which have posterior teeth with higher cusps (2.39 mm) than BT (1.67 mm), resembling more natural teeth cusps (Figures 2 and 3) [4]. Different from the BT teeth trial, in the PT trial, the patient did not perform protrusion movements.

The patient received both pairs and wore the complete dentures for two months each. While the patient was wearing the BT dentures, the involuntary protrusion movements remained. However, with the PT dentures, from the delivery session through the follow-up sessions, the maxillomandibular relation remained stable in maximal intercuspal position without traumatizing the mucosa. Harmonious occlusion and articulation were achieved without denture base dislodging during excursive movements (Figure 1(d)). According to the patient, masticatory function and retention were similar between BT and PT. Weekly follow-up sessions occurred up to 2 months. After that period, monthly follow-up sessions were set up to 1 year.

## 3. Discussion

Since the patient's old dentures were severely abraded, not only was vertical dimension decreased, but also masticatory efficiency was reduced. Mandibular displacement and alteration of the pathways that drive mastication and fatigue of the masticatory muscles are also consequences of abrasion of the artificial teeth [14, 15], which might have led to the involuntary movements of the mandible.

Both BT and PT complete dentures presented the same vertical dimension. Yet, the increase of vertical dimension itself was not sufficient to stop the involuntary protrusion movements that occurred with the old dentures and persisted with BT dentures. According to Hanau, cusp height or inclination is related to the inclination of the condylar guidance and influences the balanced occlusion of complete dentures [11]. If complete dentures are not balanced, the bases could shift during the eccentric movements and result in uneven force distribution and injure the mucosa. The selection of anatomical higher cusps artificial teeth in PT denture, closer to the patient's condylar guidance, combined with the bilateral balanced occlusion, provided a satisfactory result, since they helped the patient to maintain maximal intercuspal position and stopped the involuntary mandible movements.

Although higher cusp artificial teeth were beneficial in this case, the use of these teeth might change the direction of forces applied, as well as generating stress of greater magnitude [16]. The higher the cusps are, the more instable the prosthesis becomes, because the horizontal forces are maximized. When the residual ridge is extremely resorbed, horizontal forces tend to dislodge the denture more easily, which is negative for the patient.

Esthetics was also improved, since the higher cusps teeth were more similar to the natural teeth than the lower cusps teeth. However, this condition was only achieved due to the correct horizontal maxillomandibular record and to an accurate occlusal adjustment.

Furthermore, the chosen occlusal scheme contributed to the stability and function of the complete dentures. A lingualized balanced or a monoplane occlusion other than bilaterally balanced occlusion would not stop the patient from protruding. Selecting an occlusal scheme with higher cusps provided mandibular stability for the patient.

## 4. Conclusion

The complete dentures with higher cusps reestablished the patient's vertical dimension and masticatory function and prevented the protruding involuntary movement.
